# Workplace Learning in Clinical Laboratories: Health Professionals’ Perceptions and Practices of Training Students

**DOI:** 10.1007/s40670-026-02930-1

**Published:** 2026-10-21

**Authors:** Henriette Lorenzen, Diana Mejlstrup Barrett Momsen, Lene Gredal, Marianne Ellegaard

**Affiliations:** 1 Faculty of Health and Technology, University College Copenhagen, Copenhagen, Denmark; 2 Department of Clinical Biochemistry, Copenhagen University Hospital Hvidovre, Hvidovre, Denmark

**Keywords:** Clinical education, Clinical placement, Workplace learning, Informal learning, Student training, Health staff

## Abstract

**Introduction:**

Clinical placements, where students learn through participation in everyday work, are a central component of many health education programs, including biomedical laboratory science (BLS). While research has primarily focused on students’ experiences, less is known about how participating staff perceive their role. This study explores how BLS staff perceive and enact clinical training.

**Materials and methods:**

A qualitative study was conducted comprising four focus groups with BLS staff from two hospital settings, including both experienced and newly employed staff. Interviews were analysed using inductive thematic analysis. Activity theory guided a subsequent analysis of workplace learning tensions.

**Results:**

Six themes were identified, reflecting staff perceptions related to student training: (1) professional pride and ambassador for the field, (2) organisational aspects, (3) interpretations and perceptions of student motivation and engagement, (4) aspects of own motivation and engagement, (5) responses to perceived student engagement and methods to engage students, and (6) confidence in students, students as helpers. Staff expressed a positive commitment to supporting students and fostering professional identity. However, an Activity Theory analysis identified five interconnected tensions related to production, laboratory quality, onboarding of newly hired, division of responsibility, and pedagogical resources. These tensions shaped staffs’ opportunities to involve and support students in clinical work.

**Discussion:**

Side-by-side training is an integrated and often implicit part of everyday work. Strengthening clinical education requires recognising training as a shared organisational responsibility and creating conditions that support student participation.

## Introduction

Many education programs are dual training programs; this means that teaching and training takes place partly in scholastic settings on campus/schools and partly in internships in the practice settings of future professions. Thus, an important part of the students’ training, learning and socialization into the professions may take place in informal settings in the workplace.

Research shows that students’ experiences of clinical placement are shaped by interconnected social, pedagogical, and organisational factors [1]. Across healthcare education, inclusion in the clinical community, effective supervision, supportive relationships, and opportunities for active participation are key to learning [2, 3]. Although the literature on biomedical laboratory science (BLS) students’ perspectives of clinical education is limited, studies have highlighted that limited organisational resources may restrict students’ participation, which may hinder the development of professional identity and competencies [4–6]. However, while the conditions for this learning in practice are enacted by clinical staff who train and work alongside students in everyday practice, relatively little is known about how the staff perceive the task of guiding and supporting students.

In this study, we investigated how BLS staff perceive and navigate their role in student training and in shaping the clinical learning environment, thereby contributing to a broader understanding of how clinical placements may be further developed to support student learning. The importance of strengthening the practical part of the education is emphasized by strategies both in Denmark [7, 8] and across Europe [9, 10]. Against this background, this study examines how clinical staff perceive their responsibility for student training and discuss the opportunities and challenges associated with this role.

The aim of this paper is thus to uncover important aspects of the informal teaching environment in clinical placements of BLS students and how BLS staff perceive and approach the task of training students. The research questions are:

How do BLS staff describe and evaluate the task of training students?

What tensions emerge in relation to this task within the clinical workplace?

## Setting

The profession of BLS plays a central role in health care by performing, assuring quality, and interpreting biomedical analyses that support diagnostics, treatment, and monitoring of patients [11]. The bachelor’s degree programme in BLS in Denmark is a 3½ year education designed to qualify graduates to work independently within laboratory medicine. The programme combines theoretical teaching at the educational institution with clinical training without remuneration, which constitutes approximately one third of the total study period, corresponding to 75 ECTS [12]. Two semesters are organised as full-time clinical placements. During clinical placements, students participate in authentic biomedical laboratory practice, analysing patient samples and gaining hands-on experience through involvement in real laboratory workflows. The clinical training is organised and supervised by pedagogically trained clinical teachers, who are responsible for planning and guiding the student’s practical learning in close cooperation with the educational institution [12]. In daily practice, however, much of the students’ learning takes place through side-by-side training with BLS staff who usually do not have formal pedagogical training, but nevertheless have student training as a substantial part of their daily work. This learning environment is thus largely informal and based on workplace practice. Through this practice-based training, students may develop many of the professional competencies, practical skills, and clinical understanding required to function as a qualified BLS.

## Materials and Methods

The study employed qualitative methods in the form of focus group interviews and inductive open coding to discover emerging themes [13]. Two of the authors, who are clinical teachers, and who know the involved staff well, recruited voluntary participants. The two other authors performed the interviews. Existing professional relationships were considered, and interviews were conducted by authors without prior interaction with participants. All authors contributed to development of the research focus, research questions and formulating the interview guide. We utilized the COREQ checklist for reporting qualitative research [14]. Furthermore, our research is conducted in accordance with Danish law [15] which does not require approval by a research ethical committee for this type of research but requires participants’ informed consent (which was obtained in writing).

To include a wide variety of professional experience, data was collected through two focus group interviews for each of two locations where students undertake clinical placement: one large hospital complex and one smaller hospital approximately 10 km away. At each location, one focus group comprised BLS staff with > 10 years employment, while the other comprised more recently employed BLS staff (2–5 years). Each focus group comprised 6–8 participants (a total of 28), lasted one hour, and was audio-recorded and later transcribed.

A semi-structured interview guide was formulated to allow an open discussion between the participants, thereby capturing emerging themes [16]. The guide included three themes: (1) Purpose and rules: What do you think students should gain from the clinical placements? What are students allowed and not allowed to do? (2) Opinions and perceptions: How do you experience training of students? How do you feel about having students accompany you in your daily work? (3) Resources: What do you need in terms of training students? Is there anything you are missing in terms of training students?

All authors read the transcripts and identified preliminary emerging themes [13]. The coding was performed as an open thematic analysis [13]. First, each transcript was read and coded by two people and discussed in meeting with all four authors. During this process, an initial coding framework comprising five themes was developed and applied across all four interviews, with two authors assigned to code each interview. During this second coding step, sub-themes and thematic overlaps were identified [13]. Through iterative discussions of these [13], the coding framework was refined, resulting in six final themes. Quotations were translated from Danish into English using Google Translate and lightly edited if necessary.

Each theme was subsequently described and synthesized by one of the authors. Elements of tensions, e.g., conflicting requirements, were seen in the data, and further analysed using an activity theory lens [17].

### The Activity Theory

The activity theory assumes that human actions must be understood within their social, material, and cultural contexts. At its core is the concept of *activity*, defined as an object-oriented system of actions driven by a shared motive [17]. Activities are mediated by cultural artefacts, such as tools, technologies, and established procedures, which shape how the object of the activity is carried out. Rather than focusing on individual behaviour, activity theory emphasizes how actions and outcomes emerge through interactions within the activity system [17].

**Fig. 1:**
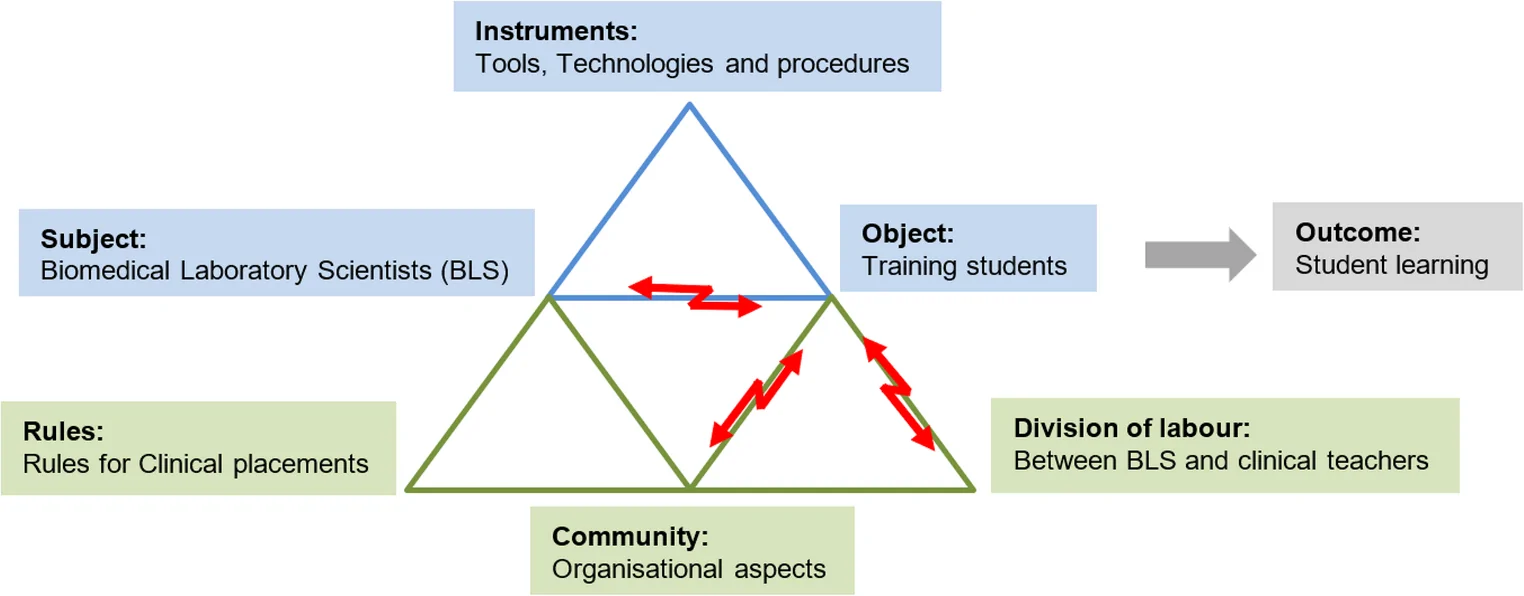
Activity system model adapted from Engeström [18], illustrating BLS training of students in clinical placement. The blue triangle represents the overarching activity-theoretical framework, while the green triangles represent structural elements of the activity system. Red arrows illustrate identified tensions between interacting elements of the activity system

An activity system comprises interconnected components: subject, object, mediating artefacts, rules, community and division of labour; how this can be visualized is exemplified in Fig. 1. These elements form a dynamic system in which changes in one component influence the others [18]. A key feature of the activity theory is its focus on contradictions, understood as tensions between elements of the system and are seen as driving forces that can lead to transformation of practice [18].

This framework was chosen, as the data indicated that side-by-side training of students in clinical practice is not an isolated task, but embedded in a complex work environment shaped by organisational demands, technologies and social relations. It allows us to examine how training is enacted within a system of interacting elements.

In a second analytical step, the themes were therefore examined in relation to the components of the activity system. Themes were linked to one or more components according to the content of the data, allowing relationships between staff, student training, organisational conditions, and the distribution of responsibilities to be examined. Tensions were identified where data indicated competing demands or conflicting requirements within or between the components of the activity system.

## Results

Through the analysis of the open coding, we sought to understand how BLS staff perceive, interpret, and enact training of students in clinical placement. We identified six overall themes (see Table 1), that show how student training is shaped by organisational conditions, staff understandings of the profession, and experiences and perceptions of students’ engagement and competencies. In analysing how these themes were connected, tensions emerged which we used the activity theory [18] to further understand. Although the participants were grouped according to their years of professional experience, meaningful differences between experienced and less experienced staff were observed only in Themes 4 and 6.

**Table 1:** Themes and illustrative quotations (Q) from focus group interviews with biomedical laboratory scientist (BLS) staff

Theme 1: Organisational aspects, tools, environment and priorities - encompasses how structural and organisational elements affect the task of training students. This includes tools such as learning objective forms (Q1), information about students (Q2) and physical constraints such as space (Q3). The theme also includes conflicting demands of, e.g., routine tasks (Q4 & 5) or onboarding new colleagues (Q6), which can collide with student training.	
Q#	Sample quotation
1	“*I like the forms. Then we know*,* this is what we are going to do*”
2	“*I think I’m missing information. How long should they be with me?”*
3	“*Often we also have one* [BLS] *in training and then we are 4* [people]* at one device … and then a couple of technicians came to visit”*
4	*“… we have deadlines where things have to run and controls have to be analysed*,* quality assurance has to be done*”
5	“*There are times when there is not much going on*,* where we do something else. We must answer an email or something*”
6	“*Of course*,* we must focus on our colleague who is new and needs to learn and quickly go on night shift*”
Theme 2: Professional pride and ambassador for the field – encompasses how staff perceive themselves as ambassadors for their clinical speciality and the BLS profession, and how they support students in discovering and developing interest in the profession, particularly as there was a preconception that their specialty is not among those that students preferred during their clinical placement (Q1), rooted in, e.g., own experiences as students (Q2). There was therefore a wish to represent the specialty well (Q3 & 4) and, more broadly, the profession (Q4).	
1	*“… students who are perhaps least interested in our specialty”*
2	*“not allowed to do anything in the 1st semester … felt that there was nothing to do*”
3	“*We have a lot of analyses and different tasks*”
4	“*I also try to be really nice to the students*,* because I think they shouldn’t have a bad experience*”
5	“*Simply teach them all these good things we do*,* and the way we say things*”
Theme 3: Interpretations and perceptions of student motivation and engagement – encompasses how staff perceive or interpret the students’ motivation and engagement. Since we have not asked the students, this theme reflects the staff’s perspectives on the students’ motivation. For example, students’ silence or passivity may be interpreted as a lack of motivation (Q1), while student engagement is found motivating (Q2). At the same time, staff emphasise their own role in activating and motivating students (Q3, 4 & 5), and it is acknowledged that lack of opportunities to participate can be demotivating (Q6). Some staff, question whether some students should have chosen the BLS field (Q7).	
1	“*It’s a bit difficult when they just sit and are silent and don’t say much. And no matter what you say. Did the person understand? Yes*,* it is difficult to motivate if they don’t have the motivation*”
2	*“… great that they are so new and curious*,* that they make you think”*
3	“*And they* [an initially insecure student] *become more and more terrified and hardly dare. If little by little during the first days they are allowed to do some things* [they become more active]”
4	“*It is also on our shoulders to assess how much we can move this person*,* right?*”
5	“*You* [students] *have to have some successes”*
6	“*Someone who just sits on the sidelines for a whole week and just sinks further and further down into their chair. Well*,* no one wants that”*
7	*“They shouldn’t choose the BLS field at all*,* many of them”*
Theme 4: Aspects of own motivation and engagement - covers staffs’ own commitment and motivation in involving students in the tasks. Overall, staff express a positive attitude (Q1 & 2) and describe students as contributing to professional reflection (Q3). However, involving students can also be experienced as demanding (Q4). Students who remain passive without asking questions or actively participating may reduce staff motivation to engage (Q5). A perceived lack of pedagogical skills can also challenge motivation (Q6), which to some extent can be mitigated by feedback from clinical teachers (Q7 & 8). Motivation may be influenced by staff’s own level of experience, with less experienced staff describing difficulties relating to students (Q9 & 10). Among experienced staff, students who are perceived as committed, quickly acquire new knowledge (Q11) and contribute to the workflow (Q12) promote motivation.	
1	“*I think many of us find it really nice to have them with us”*
2	“*I actually don´t think I have ever had a bad experience with a student*”
3	“*they make you think*”
4	“*mentally draining*”
5	“*I didn’t really know how much they benefitted from it*”
6	*“not pedagogically inclined”*
7	“*I also want to know if I did it well enough or if I could change something*”
8	“*When you *[staff]* get that feedback*,* you want to do it just as well the next time you work with students*”
9	“*I find it a bit difficult to relate to the student if I’m new to it myself*”
10	“*I also tell the student that I can’t answer and that I’m new myself*”
11	*“If the student is eager to*,* and wants to learn something*,* then it’s actually great*”
12	“*It’s like having a new colleague*”
Theme 5: Responses to perceived student engagement and methods to engage students - concerns how staff react to the situations described in themes 3 and 4. Drawing on, e.g., their own experiences as former students (Q1), staff highlight the meaningfulness of students participating in practice alongside practicing professionals (Q2), and even how they nudge students to participate (Q3 & 4), while also acknowledging that finding suitable tasks can be challenging (Q5). They also stress the importance of dialogue with students. This includes debriefing after mishaps (Q6), but also general talk (Q7), and the positive effects of establishing a good relationship (Q8 & 9).	
1	“*… when the person* [BLS] *you were with kind of showed interest in you … he was good at explaining things and then let you try several things*”
2	“*Get them to do something instead of just sitting. It is so important to involve them as much as possible*”
3	“*I am probably one of those who pushes*”
4	“*You say: You just feel*,* you just see what it´s like …then they *[students]* will feel it themselves … well*,* I would like to try that one* [vein], *that is big*”
5	“*I don’t know what to come up with … I know they are bored sometimes*”
6	*“How was this? It is important to say*,* hey*,* yes*,* it can happen to anyone. And explain it in a constructive way instead of being negative and scolding. No one gets anything out of it. And it’s also about appreciating it afterwards*”
7	*“…kind of try to start a conversation with them*,* just a normal conversation. Sometimes they open up*”
8	“*You joke and laugh. It’s fun for everyone*,* it’s going to be a good day*”
9	“*Well*,* for me it’s important that they have a good experience*,* that they feel like they’re welcomed*,* and that we want them*”
Theme 6: Confidence in students, students as helpers – concerns staff’s confidence in entrusting students with tasks. Staff’s confidence in students’ abilities and in their own ability to address potential student mistakes varies. Some staff are comfortable allowing students to try a range of tasks (Q 1 & 2), while others are more cautious based on previous experiences with students (Q3 & 4) and the precision required for certain tasks (Q5). Experienced staff describe greater confidence in entrusting students with tasks (Q6). Some describe the mutual trust as leading to positive experiences (Q7, 8 & 9).	
1	“*They are actually allowed to do a lot*,* but under my supervision of course*”
2	“*Sometimes they can operate the devices themselves*”
3	*“I’ve just experienced that it’s gone wrong many times. I just don’t want my name on it*”
4	“*I have had some experiences… where the patient sample had to be retaken*”
5	*“Something that really requires precision*,* I would not let them”*
6	“*I feel like I’m so experienced*,* so I let go a lot because I will be able to quickly solve problems afterwards”*
7	“*She was super proactive and she wanted to do things … we went through everything together*”
8	“*He helped me so much that day*,* and it’s like having a new colleague*”
9	“*They are there sometimes for several days at a time*,* so they are almost autonomous*,* and that also benefits me*”

The analysis points to an overall positive commitment among staff to supporting students and integrating their training into everyday clinical practice. Few differences were identified between less experienced and experienced staff, with differences mainly relating to their own motivation and confidence in entrusting students with tasks. At the same time, the findings indicate tensions between providing opportunities for student training and the demands of clinical practice. Thus, student training is embedded in a work environment in which different needs, expectations and priorities have to be balanced. These tensions were further examined in relation to the components of the activity system.

### An Activity-theoretical Analysis of Competing Objects

Drawing on an activity theoretical framework [18] (see Fig. 1), the BLS is conceptualised as the *subject* and training of students as the *object*, mediated by *instruments*, such as technologies, and procedures that enable student learning. The activity is further shaped by *rules* for clinical placement, the *community* understood as organisational aspects, and the *division of labour* between BLS and clinical teachers.

The six themes describe the main empirical pattern, while the activity theoretical analysis identified five interconnected tensions in student training. These concerned competing priorities between (1) laboratory production and student training, (2) student participation and laboratory quality, (3) student training and onboarding new staff, (4) student support and pedagogical resources, and (5) student training and division of responsibility between staff and clinical teachers.

First, a tension emerges between organisational aspects related to laboratory production (*community*) and the responsibility for training students (*object*) (see Theme 1 & 5). The staff articulate a clear training ideal: “[A student] *who just sits on the sideline for the whole week and sinks deeper and deeper into the chair*,* well*,* nobody wants that*,* so it´s important to get the ball rolling or at least say why we don´t have time right now*”. However, this ideal is challenged under conditions of high workload: “*There is total pressure … then I think none of us can have students*” and “*We constantly think about production and then it´s sometimes hard to give time and engagement to motivate a student who doesn´t want to*”. Thus, the object of student training competes with the production demands, limiting staff’s opportunities to enact their responsibility for student learning.

Second, a related tension emerges between student training *(object)* and staff’s responsibility for carrying out laboratory work accurately *(subject)* (see Themes 3, 5 & 6). Staff describe how they limit which tasks students can perform when they consider that it *“requires precision”*, particularly based on previous negative experiences: *“it’s gone wrong many times”.* Thus, opportunities for student participation are balanced against staff responsibility for ensuring quality of laboratory work.

Third, a tension emerges between student training (*object*) and the organisational aspects related to onboarding of newly hired colleagues (*community*) (see Theme 1). Staff describe competing demands for time and attention: “*Who do I ignore – the new employee or the student?*”. Both forms of training are integrated into daily routines and competes for staff’s resources. This creates a particular challenge for less experienced staff, who describe difficulties in training students while still undergoing their own training: “*It affects your own training*” while another describes it as “*difficult to relate to the students when I am new myself*”. Thus, these situations position staff in dual roles as learners, and supervisors, creating tensions within their subject position of the activity system.

Fourth, a tension emerges between the expectation that staff support students training (*object*) and staff’s perceived pedagogical resources (*subject*) (see Theme 4). Some staff describe themselves as lacking pedagogical competencies. Students are at times perceived as disengaged, as reflected in statements such as: “*You can see by looking at them that they don´t want to*”. This perception appears to influence the level of support provided, which may depend on the student’s visible engagement: “*If they don´t show interest*,* I just say: The device can do this*,* and if they show interest*,* it will be elaborated.*” Thus, the expectation to support student training conflict with staffs perceived pedagogical resources and their responses to students’ engagement.

Finally, a tension emerges between student training (*object*) and the distribution of responsibility for the students between staff and clinical teachers (*division of labour*). Staff describe situations where responsibility for students is unclear: “*Sometimes we really don’t know what to do with a student … they *[clinical teachers]* could step in a little more for the students’ sake*”. Others describe difficulty balancing training and production responsibilities: *“There is still something called the teachers’ task and staffs’ task: “I think it is sometimes difficult to navigate … of course you want to do a lot for a student*,* but we are constantly thinking … we have a whole production”.* Thus, these tensions reflect ambiguity in how training responsibilities are shared and enacted between BLS staff and clinical teachers.

Taken together, these tensions show how student training is shaped by competing demands related to laboratory production, laboratory quality, onboarding of new colleagues, pedagogical resources, and the distribution of training responsibilities.

## Discussion

This study explored how BLS staff experience and enact side-by-side training of students during clinical placement. The findings show that staff contribute to shaping students’ clinical learning, and that this role is enacted within a complex work environment where professional responsibilities, and educational expectations intersect.

A central finding is that staff perceive themselves as ambassadors for the profession. This sense of professional pride motivates them to provide students with positive experiences. The desire to “*Simply teach them all these good things we do*,* and the way we say things* ” suggests that training can be understood as a form of professional identity work. By introducing students to everyday practices, staff invite students into the clinical community. Drawing on a Community of Practice perspective [19], this can be understood as supporting students’ movement from peripheral to more central forms of participation and contributing to the development of professional identity through engagement with the shared practices, language, and culture of the profession. This aligns with findings from studies on medical students showing that clinical staff are often positively motivated to engage in teaching and see it as a part of their professional role [20, 21].

In this study, staff perceive training as professionally meaningful, as students may stimulate reflection, refresh existing knowledge, and sometimes contribute to the workflow. Similar findings have been reported in studies of other health professions, where students are described as keeping staff professionally alert and reflective [20–22].

When students demonstrate initiative and competence, they may be perceived almost as colleagues, which can further strengthen staff’s motivation to engage in training - an effect also observed in nursing clinical placement [22]. However, the findings also show that staff may interpret student silence or passivity as lack of motivation. At the same time, they acknowledge that students may initially be insecure and that gradual participation may increase engagement, a pattern also reported in nursing contexts [22]. This raises the possibility that hesitant students may receive fewer opportunities to participate if their behaviour is interpreted as a lack of motivation, potentially reinforcing their disengagement. From a Workplace Learning Theory perspective, this suggests that access to learning is shaped by the interplay between workplace affordances, such as norms and practices, and individual engagement [23]. Consequently, opportunities for participation are not equally distributed but continuously negotiated, depending on how students are perceived and on the priorities of the workplace.

We used Activity Theory to situate side-by-side training within a broader organisational context [17], highlighting how five interconnected tensions shape training practices and opportunities for student participation.

The coexistence of clinical laboratory production and educational responsibilities represents a central tension, as training is integrated into ongoing work rather than organised as a separate activity. While staff emphasise an ideal of involving students actively in tasks and preventing them from becoming passive observers, this ideal is difficult to realise in clinical laboratories operating under continuous workflows and strict deadlines. A related tension identified in the study concerns competing demands of training students and onboarding newly hired colleagues, further illustrating how multiple demands for staff time can arise within the clinical workflow. From a Workplace Learning Theory perspective, this tension reflects how affordances for learning are shaped by the primary goal of maintaining service delivery [23]. Increasing workload tends to make pedagogical engagement secondary for staff, reducing opportunities for student participation and potentially affecting the quality of training [20]. This tension between service delivery and teaching has also been documented in clinical education across other health professions [20, 24–26], with clinical placement by staff sometimes experienced as a ‘burden’ [21]. However, from a Situated Learning perspective [19], learning is inherently embedded in social practice and cannot be separated from work activities. From this perspective, production demands should not simply reduce time for training but rather shape the conditions under which training occurs.

This tension is closely connected to the division of responsibility between staff and clinical teachers. Staff describe situations in which it was unclear when clinical teachers should intervene and what responsibility should be assumed by staff. Thus, unclear responsibilities may add to the challenges of balancing educational and production demands. Clearer dialogue about roles and responsibilities may therefore help staff navigate the educational demands of clinical work.

We identified a tension between student participation and laboratory quality. Participation depends on entrustment, a dynamic and context-dependent process in which supervisors continuously adjust students’ level of responsibility based on observed competence, reliability, and patient safety considerations [27]. In our study, trust in students appeared to develop gradually through situated experiences of competence and responsibility. Drawing on a Community of Practice perspective [19], these findings suggest that BLS staff continuously negotiate students’ legitimate participation in clinical practice by balancing participation with concerns related to quality assurance.

More experienced staff appeared more comfortable in allowing students to perform tasks, while some less experienced staff found training more challenging due to lower confidence in overseeing students. This may create a further tension when staff themselves are developing their own professional competencies while also being expected to support student training.

The variability in BLS staff’s confidence in their pedagogical competencies also reflects a tension between the expectation to support student training and the pedagogical resources available to staff. Some describe themselves as lacking pedagogical training or feeling uncertain about whether students benefit from their training. Findings from other health professions show that these challenges are intensified in unpredictable clinical environments [24, 25]. This points to a potential gap between expectations placed on staff to support student training and the pedagogical resources available to them. Research from other health professions similarly shows that effective training requires competencies beyond disciplinary expertise, including knowledge of pedagogical strategies, the ability to adapt to students at different levels [24, 25, 28] and feedback practices [28].

These findings highlight a need to strengthen pedagogical competencies among staff. Given that side-by-side training of both students and colleagues is used across health professions, relevant educational objectives may be integrated into education programs. Future research may explore which pedagogical competencies are most relevant for clinical staff involved in side-by-side training and how organisational and pedagogical support may influence students’ opportunities for active participation in clinical practice. Supporting strong feedback cultures is explicitly pointed to as a possible way to do this.

### Limitations

As this is the first study of the practice learning setting in BLS from the point of view of the practitioners, it represents a first step in illuminating this practice. While we have attempted to include a wide variety of perspectives in our recruitment for the focus groups it still represents a limited sample and caution should be used when applying to different contexts.

## Conclusion

Taken together, the findings suggest that side-by-side training of students in clinical departments should be understood as a collective and organisational responsibility rather than solely an individual task. Clinical departments should recognise student training as an integral part of clinical work and support staff in balancing production and educational responsibilities. Staff may further benefit from access to pedagogical support, as well as opportunities for dialogue with clinical teachers, and making feedback on supervisory practices an explicit priority. Finally, creating conditions that facilitate student participation also appears crucial. Even small opportunities for active involvement may help prevent the disengagement, that respondents describe can occur when students remain observers for extended periods.

## Data Availability

Data generated and analysed during this study are included in this article. Additional data are available on reasonable request from the corresponding author. Restrictions apply to these data due to privacy and ethical considerations.
